# Identifying inaccuracies on emergency medicine residency applications

**DOI:** 10.1186/1472-6920-5-30

**Published:** 2005-08-16

**Authors:** Eric D Katz, Lee Shockley, Lawrence Kass, David Howes, Janis P Tupesis, Christopher Weaver, Osman R Sayan, Victoria Hogan, Jason Begue, Diamond Vrocher, Jackie Frazer, Timothy Evans, Gene Hern, Ralph Riviello, Antonio Rivera, Keith Kinoshita, Edward Ferguson

**Affiliations:** 1 Division of Emergency Medicine, Washington University in St. Louis, St. Louis, MO, USA; 2 Department of Emergency Medicine, Denver Metro Health Center, Denver, CO, USA; 3 Department of Emergency Medicine, Pennsylvania State University, Hershey, PA, USA; 4 Division of Emergency Medicine, University of Chicago, Chicago, IL, USA; 5 Department of Emergency Medicine, Indiana University School of Medicine, Indianapolis, IN, USA; 6 Department of Emergency Medicine, NewYork-Presbyterian Hospital, New York, NY, USA; 7 Department of Emergency Medicine, University of Alabama, Birmingham, AL, USA; 8 Department of Emergency Medicine, Medical College of Virginia, Richmond, VA, USA; 9 Department of Emergency Medicine, Highland General Hospital, Oakland, CA, USA; 10 Department of Emergency Medicine, Thomas Jefferson University, Philadelphia, PA, USA

## Abstract

**Background:**

Previous trials have showed a 10–30% rate of inaccuracies on applications to individual residency programs. No studies have attempted to corroborate this on a national level. Attempts by residency programs to diminish the frequency of inaccuracies on applications have not been reported. We seek to clarify the national incidence of inaccuracies on applications to emergency medicine residency programs.

**Methods:**

This is a multi-center, single-blinded, randomized, cohort study of all applicants from LCME accredited schools to involved EM residency programs. Applications were randomly selected to investigate claims of AOA election, advanced degrees and publications. Errors were reported to applicants' deans and the NRMP.

**Results:**

Nine residencies reviewed 493 applications (28.6% of all applicants who applied to any EM program). 56 applications (11.4%, 95%CI 8.6–14.2%) contained at least one error. Excluding "benign" errors, 9.8% (95% CI 7.2–12.4%), contained at least one error. 41% (95% CI 35.0–47.0%) of all publications contained an error. All AOA membership claims were verified, but 13.7% (95%CI 4.4–23.1%) of claimed advanced degrees were inaccurate. Inter-rater reliability of evaluations was good. Investigators were reluctant to notify applicants' dean's offices and the NRMP.

**Conclusion:**

This is the largest study to date of accuracy on application for residency and the first such multi-centered trial. High rates of incorrect data were found on applications. This data will serve as a baseline for future years of the project, with emphasis on reporting inaccuracies and warning applicants of the project's goals.

## Background

The residency application process is predicated on the validity of the credentials submitted by an applicant. Previous studies, however, have found that 10–30% of applications contain errors. [1-6] In emergency medicine (EM), Roellig, et. al. showed that 13.3% of applicants to a single EM residency had at least one error on their application, and 4% had more than one.[7] Rates of erroneous claims were similar for claims of authorship (21.3% erroneous), Alpha-Omega-Alpha (AOA) claims (35.7%) and advanced degrees (26.7%). In a previous single center investigation, Gurudevan, et. al. reported that 20.4% of applicants who claimed authorship of a peer- reviewed paper had at least one error in their reference.[8]

Presently, the residency program discovering misrepresentations may only act on the information internally. Discussion of applicants among programs could be viewed as violating the ethics and rules of the National Residency Matching Program (NRMP) match, thus, programs are reluctant to share information. If errors are identified, corrective action can be implemented either by contacting the Dean's Office of the applicant's medical school or the NRMP. Further fact-checking and any disciplinary action is then the responsibility of the notified organization. In addition, ongoing anti-trust litigation (*Jung vs. Association of American Medical Colleges*) against the NRMP, residency review committees (RRC's), Accreditation Council for Graduate Medical Education (ACGME), etc. makes a coordinated fact-checking effort by any one agency unattractive.

To date, there have been no published reports of attempts to identify or impact the inaccuracy rate. Our study group was formed with the intent of documenting inaccuracy rates in publications, AOA claims and advanced degrees at multiple centers, and to seek ways of impacting the problem on a national level. This multi-phased study attempts to characterize the magnitude and characteristics of application inaccuracies while later stages will attempt to impact the error rate.

## Methods

This is a prospective, multi-center, single-blinded, cohort study of applicants to EM residency at involved residency sites. Each study site obtained permission from their Human Studies Committee (or equivalent) prior to beginning the project. All inclusion criteria, exclusion criteria, endpoints and methods were prospectively defined. Applicants were not made aware of the study prior to investigation. Programs invited to participate were asked to keep the existence of the study confidential from their own students to avoid contamination of the applicant pool.

The applications of all residents applying to participating residency programs were eligible for analysis. Participating sites randomly reviewed 10% of their applicant pool or a minimum of 50 applications. Randomization was achieved by assigning each site a single-digit number. The site then reviewed all ERAS applications whose unique application identifiers ended with that number. If the number of applications at a given site did not meet the minimum of 50 applications, applications with unique identifiers ending with the next higher digit were reviewed. Additionally, each site had the opportunity to review other applications at their discretion without regard to randomization as long as they completed a minimum of 50 randomized reviews. Incomplete applications and those from medical schools not accredited by the United States Medical Licensing Examination were excluded from the analysis.

All study data was recorded on a secure, encrypted, internet database. The only personal identifier entered was the NRMP number. Once this was entered, the database irreversibly converted it to a unique study number by a fixed, but random, formula. This allowed for calculation of inter-rater reliability, while blinding investigators to the identity of the applicants.

Peer-reviewed publications were verified by searching at least two publication databases (Medline, PubMed, etc.) or review of the referenced journal. If either of these methods identified the publication in question, it was considered "verified". Applicants could also be asked to supply a copy of the publication or submission. Publications cited as being "submitted" or "in press" were excluded from analysis. Publication errors were classified as those which claimed improper order of authorship, journal citation, or those which could not be found with the above techniques. Each publication could have more than one error.

AOA status was verified by contact with the national headquarters of Alpha Omega Alpha (computer search of members) or by review of the applicant's Medical Student Performance Evaluation (MSPE) or dean's letter of recommendation.

Advanced degrees were confirmed by the awarding institution. If the degree was earned concurrently with medical training, comments in the MSPE or dean's letter were considered evidence of accuracy.

The assessed endpoints are listed in table 1.

**Table 1:** Initial results

	Number	%	95% CI (%)
**Peer-reviewed publications claimed**	**256**		
Accurate	151	59.0	53.0–65.0
Inaccurate	101	41.0	35.0–47.0
Incorrect author placement	7	6.0	0.0 – 14.6
Unconfirmed	40	34.5	25.8 – 43.2
Errors of publication type	52	44.8	36.2 – 53.4
"Benign" errors	8	7.6	2.5 – 12.7
Other error in peer-reviewed journal	17	14.7	6.0 – 23.4
**Claims of AOA membership**	**47**	**9.5**	**6.9 – 12.1**
AOA Status verified	47	100	99.1–100
**Advanced degree claimed**	**51**	**10.3**	**7.6 – 13.0**
Accurate	22	43.1	29.5–56.7
Inaccurate	7	13.7	4.4 – 23.1
Unable to be confirmed	22	43.1	29.5 – 56.7

An error could be classified as "benign," if it was felt that the error could not possibly be due to malicious intent on the part of the applicant and could not benefit the application. Two examples of benign errors include typographical errors and incorrect page or journal numbers in a reference. Judgment about intent was neither made nor implied on any other misrepresentation.

For all non-benign errors, the programs were to inform the applicant's Dean's office and the NRMP of the error, to allow for corrective action. If an applicant was made aware of a concern about his or her credentials, the applicant could verify the claim with appropriate documentation. Notification of the applicant was not required by the study protocol, but was allowed.

## Results

A total of 493 applications were screened (28.6% of all applicants submitting at least one ERAS application to an EM residency program). 56 applications (11.4%, 95% CI 8.6–14.2%) contained at least one inaccuracy. Eight of these were judged to be "benign" leaving 48 applications with a non-clerical error (9.8%: 95% CI 7.2–12.4%). The reviews were conducted at nine residencies. Please refer to table 1 for detailed results.

Thirty-three applications (6.7%) were screened by two study centers. Only 2 (6.1%, 95%CI 0.0–14.3) had disagreeing data. In one of these disagreements, one reviewer classifying an error as a "benign," while another reviewer did not record the error. A kappa could not be calculated as the total number of reviewers involved in these 33 cases was not tracked.

The 493 applications referenced 737 publications (mean 1.49 per applicant, range 0–12). Of these, 256 (34.7%, 95%CI 31.3–38.1%) were from peer-reviewed journals. Errors were identified on 105 of these (41.0%, 95%CI 35.0–47.0%).

Fifty-one applicants (10.3%, 95%CI 7.6–13.0%) claimed advanced degrees. Of these, seven (13.7%, 95%CI 4.4–23.1%) were inaccurate. However, 22 (43.1%, 95% CI 29.5–56.7%) claims could not be verified as either accurate or inaccurate. If these are excluded from analysis, 7/29 (24.1%, 95%CI 8.5–39.7%) of claims were inaccurate.

Due to a database flaw, it was not possible to accurately monitor how often the NRMP or Deans' Offices were notified. There was general agreement amongst sites that there had been reluctance to pursue inaccuracies. No site reported a corrected application being submitted by the NRMP. It was not possible to reliably track the actions taken by the deans' offices.

## Discussion

This study represents the largest and only multi-centered study to date of inaccuracies on residency applications. Review of 28.6% of the total applicant pool revealed an error on 11.6% of all applications. This is similar to those described in prior reports in EM as well as other specialties [1-8]. If benign errors are excluded, the rate of major errors on applications is nearly 10%, which would place EM among the lowest of reviewed fields.

Our study design was mandated by features of the application system. For example, there is no centralized way to review applications, so a multi-centered trial was necessary, despite the duplication of effort this entails. In addition, the rules governing the behavior of programs during the NRMP match make discussion of applicants between residencies difficult, so programs are often reluctant to share negative information about applicants. This forced our study towards a design that would maximize protection of the applicant, while utilizing the available pathways for reporting of errors and for corrective action. In addition, ongoing legal efforts necessitated legal consultation with a lawyer assigned to the defense of *Jung vs. Association of American Medical Colleges *in order to ensure protection of the residency programs involved.

A prior retrospective study at one of the authors' institution (EDK) showed similar findings with a few notable differences. We found no erroneous AOA claims while Roellig[7] found an error rate of 35.7%. It is possible that this is due to the differences in included applications: the prior study included osteopathic students while ours did not. As three of the five erroneous AOA claims in that study were from osteopathic students, it is possible that this was due to students attributing this moniker to the American Osteopathic Association.

Secondly, our study found a higher rate of errors on publications (41.0% vs. 21.3% in Roellig[7] and 20.4% in Gurudevan[8]) than previously reported. There are many conceivable sources for this discrepancy. It is possible that the literature search methodology was insufficient in our project, as it relied on review by database, rather than journal review. Before further study is begun, time from publication to database entry must be assessed. In addition, the prior, retrospective, studies were able to assess journals listed as "in press," which was not possible with a prospective design, due to the time interval between acceptance, revision and publication. The inter-rater reliability implies that the methodology provided consistent results, but the disparity between our results and previous results from our field is concerning.

A third difference is that the prior study[7] found 73.3% of advanced degree claims to be verifiable while we were able to verify only 56.9%. Several institutions contacted by the authors had procedures that prevented verification. The previous, retrospective, trial could invest the time necessary to address these procedures, while a prospective, multi-centered trial could not.

Lastly, during the Roellig trial[7], we were able to confirm the AOA status of applicants up to 8 months after the application season. During the current project, the AOA offices were less able to accommodate our inquiries. Should this project become much larger, the AOA staff may become reluctant to verify applicant election to AOA. However, the MSPE's were uniformly helpful in confirming AOA status. It should be noted that we chose not to validate claims of AOA nomination as that process is more loosely defined, and other than each applicant's dean's office, there is not a reliable avenue for verification (the AOA database does not track nominations).

In our study, members of the research program did not reliably take corrective action when an inaccuracy was found. Because of this, we are unable to draw conclusions as to the efficacy of the reviews. If this persists, it may limit the effect of the project. This matter will be strongly emphasized before the next application season.

We did not attempt to assess whether the inaccuracies were intentional though we did attempt to differentiate "benign" and significant errors. For instance, typographical errors may be reasonably assumed to by unintentional whereas an erroneous publication or advanced degree claim is more likely assumed to be intentional. Others inaccuracies may be more difficult to assess. For instance, the misrepresentation of the order of authorship of an article could be assumed to be intentional or simply due to a lack of understanding regarding the significance of the order.

Since the interpretation of motivation is fraught with difficulty, the study did not make any recommendations for how each program used the findings on its applicants. In addition, we did not seek to recommend specific action by any dean's office or the NRMP. Decisions of this nature were outside our scope of research, but may prove to be fertile ground for future investigation.

During the next application season, we intend to expand our study to other sites, with a goal of eventually reviewing the majority of applicants to our specialty. In addition, we will publicize the existence of the trial to the applicants before their applications are submitted. In future studies, we aim to impact the number of erroneous claims. By notifying applicants' institutions and the NRMP regarding inaccuracies and publicizing these efforts, we anticipate a decrease in intentional erroneous claims on applications.

## Limitations

Though multi-centered, this study was not large enough to review the entire NRMP EM applicant pool. Nevertheless, by randomly reviewing nearly 29% of all applications we feel the sample size is adequate to draw general conclusions regarding the error rate on these applications. The time-constraints of the match process limits review of some data (manuscripts referenced as "in press," advanced degrees, etc.) Future studies will assess the impact of specific interventions on the error rate.

## Conclusion

We report the findings in the first year of a 3-year project aimed at assessing and impacting inaccuracies in residency applications in Emergency Medicine. 11.4% of applications had at least one error and 9.9% had at least one non-clerical error. Publication claims were found to contain errors in 41% of cases. Though at times difficult to verify, erroneous claims on advanced degrees were made in 14 – 24% of applications. All AOA claims were verified.

## Competing interests

The author(s) declare that they have no competing interests.

## Authors' contributions

All authors read the manuscript and approved the final manuscript.

EK was involved in study concept and design, data acquision, analysis and interpretation, drafting of the manuscript and statistical analysis.

LS was involved in study concept and design, data acquision, analysis and interpretation, and critical review of the manuscript.

LK was involved in study concept and design, data acquision, analysis and interpretation, and critical review of the manuscript.

DH was involved in study concept and design, data acquision, analysis and interpretation, and critical review of the manuscript.

JT was involved in data acquision, analysis and interpretation, and critical review of the manuscript.

CW was involved in study concept and design, data acquision, analysis and interpretation, and critical review of the manuscript.

OS was involved in study concept and design, data acquision, analysis and interpretation, and critical review of the manuscript.

JB was involved in data acquision, analysis and interpretation, and critical review of the manuscript.

DV was involved in study concept and design, data acquision, analysis and interpretation, and critical review of the manuscript.

JF was involved in data acquision, analysis and interpretation, and critical review of the manuscript.

TE was involved in study concept and design, data acquision, analysis and interpretation, and critical review of the manuscript.

GH was involved in study concept and design, data analysis and interpretation, and critical review of the manuscript.

RR was involved in study concept and design, data acquision, analysis and interpretation, and critical review of the manuscript.

AR was involved in data acquision, analysis and interpretation.

KK was involved in study concept and design, data acquision, analysis and interpretation.

EF was involved in study concept and design, statistical expertise and technical support.

## Pre-publication history

The pre-publication history for this paper can be accessed here:


